# Structural Integrity Assessment of Composites Plates with Embedded PZT Transducers for Structural Health Monitoring

**DOI:** 10.3390/ma14206148

**Published:** 2021-10-16

**Authors:** Tianyi Feng, M.H. Ferri Aliabadi

**Affiliations:** Structural Integrity and Health Monitoring, Department of Aeronautics, Imperial College London, South Kensington, London SW7 2AZ, UK; m.h.aliabadi@imperial.ac.uk

**Keywords:** structural health monitoring (SHM), thick composites, embedded PZT transducers, fatigue tests, tensile and compressive tests

## Abstract

Active sensing using ultrasonic guided waves (UGW) is widely investigated for monitoring possible damages in composite structures. Recently, a novel diagnosed film based on a circuit-printed technique with piezoelectric lead zirconate titanate (PZT) transducers has been developed. The diagnostic film is a replacement for the traditional cable connection to PZT sensors and has been shown to significantly reduce the weight of the host structure. In this work, the diagnosed films were embedded into composite structures during manufacturing using a novel edge cut-out method during lay-up, which allowed for edge trimming after curing. In this paper, the effect of fatigue loading on the integrity of PZT transducers is initially investigated. The electro-mechanical impedance (EMI) properties at different fatigue loading cycles were used as the diagnostic measure for the performance of the sensors. At the same time, the behaviours of UGW were investigated at different fatigue loading cycles. It was found that the EMI properties and active sensing behaviours remained stable up to 1 million cycles for the force ranges of 0.5~5 kN and 1~10 kN. Next, the effect of embedding the diagnosed film on the mechanical properties of the host composite structure was investigated. Tensile and compressive tests were conducted and the elastic modulus of composite coupons with and without embedded PZT diagnosed films were compared. The elastic modulus of composite coupons with PZT diagnosed films embedded across the entire coupon reduced by as much as 20% for tensile tests and just over 10% for compressive tests compared to the coupons without embedded sensors. These reductions are considered the worst-case scenario, as in real structures the film would only be embedded in a relatively small area of the structure.

## 1. Introduction

Currently, composite materials are widely used in the manufacturing of airframes due to their high mechanical performance with lower densities and high resistance to fatigue and corrosion [1,2,3,4,5]. However, they can suffer from unforeseen impact events during their operation and maintenance, which may result in barely visible damages. Structural health monitoring (SHM) is a promising nondestructive inspection technique which offers a real-time damage detection under operational and environmental conditions for aircrafts [6,7,8,9]. There are many different types of sensors that can be used in SHM, such as strain gauges [4], optical fibres (OF) [10], carbon nanotube (CNT) sensing networks [11], and piezoelectric lead zirconate titanate (PZT) transducers [12]. These sensors are normally installed on the surface of composites but can also be embedded inside the host structure.

Surface-mounted sensors can be easily damaged during routine maintenance if not protected by additional protective layers which in turn add weight to the overall structure. In addition, for thick composite parts, PZT transducers become less sensitive to internal damage such as delamination [13]. Therefore, embedding transducers into thick composite structures can be a desirable way of alleviating the above difficulties. On the other hand, any inclusion during composite manufacturing may cause material degradation and affect the strength [14]. Hence, it is important to evaluate the effects of embedded transducers on the mechanical properties of the host composite structures.

For strain gauges, Belhouideg et al. [4] conducted mechanical tests (tensile, three-point bending, fatigue tests) when embedding strain gauge into glass fibre reinforced epoxy composites for SHM techniques. They demonstrated that reductions of tensile tests with embedded strain gauges for the tensile stiffness and tensile strength were 5.8% and 1.5%, respectively. Furthermore, the three-point bending test results showed that the flexural strength and stiffness increased by 1.5% and 5.5%, respectively, with embedded strain gauges.

There are many research papers published focusing on the study of mechanical properties when embedding optical fibres into composites for SHM techniques. Ruzek et al. [10] investigated the compressive behaviour of composite fuselage panels with embedded fibre Bragg grating (FBG) sensors. The results showed that the embedded FBG sensors could not provide the correct information about maximum local deformation or detect notable strains due to their placement on the neutral axis. Karpenko et al. [15] studied the fatigue behaviour by embedding FBG sensors in the bond-line of a composite lap-joint. They demonstrated that the spectral response of the embedded FBG sensors was sensitive to fatigue loading and damages. Kocaman et al. [16] evaluated heat generated during the fatigue process and demonstrated that increasing temperature and local strain data could be used to monitor the internal damage of glass fibre composites by using embedded FBG sensors. Other studies related to mechanical properties of composites with embedded FBG sensors for SHM techniques were conducted by Li et al. [17]. The fatigue test results showed that the sensing performance remained good after two million cycles. In addition, the tensile test results demonstrated that the effect of embedded FBG sensors to composites was negligible. Karatas et al. [18] studied the fatigue behaviour of composites with embedded FBG sensors. They demonstrated that the embedded FBG sensors were more durable than the surface-mounted FBG sensors. Souza et al. [19] proposed a novel embedded technique based on vacuum assisted resin infusion (VARI) where they embedded optical fibre (OF) sensors in three differences interfaces. They found that embedded OF sensors performed differently depending on their infusion qualities and surroundings during tensile tests.

For carbon nanotubes (CNT), Nisha et al. [11] studied the effect of tensile properties on embedding CNT into glass fibre reinforced polymer (GFRP) materials. They found that the embedded CNT presented the same tensile properties compared to those without embedding. In addition, Aly et al. [20] introduced a novel embedding CNT technique for laminated composite structures to improve the sensibility, stability, and repeatability of CNT sensing layers. The mechanical test results showed that composite coupons with embedded CNT sensing networks exhibited linearity in tension and nonlinearity in compression. Furthermore, Ahmed et al. [21] proposed a novel technique for composite structural repair of fatigue cracks where they embedded a CNT-based layer in the bond-line between the fatigue crack substrate and carbon fibre composites. They found that the piezoresistive behaviour of the CNT sensing layer could still be measured, and the structural/sensing repair could extend the fatigue life by 380%. More recently, Bekas et al. [22] printed a planar interdigital capacitive sensor directly onto the surface of a composite lap joint to determine the initial quality of curing the bonded composite joints and assessing their long-term durability. Their reported results from the mechanical characterisation indicated that the developed sensor did not affect the quality of the bond-line, while the added weight of the sensor was deemed negligible.

For PZT transducers, Lin et al. [12] tested the mechanical properties of embedding “SMART Layers” (Stanford multi-actuator-receiver transduction layers) into composites for SHM purposes; the results showed that the embedding of SMART Layers in an isolated section and small parts related to the overall structure did not degrade the structural integrity of the host composite structures. Masmoudi et al. [14,23] embedded the PZT transducers into thick GFRP materials and mechanical tests showed there were no differences for GFRP materials with and without embedded PZT transducers. Tuloup et al. [24] embedded PZT transducers into polymer-matrix composites (PMC) and found that the mechanical resistance and maximum longitudinal strain increased by 6% and 11%, respectively. Furthermore, reductions of the elastic modulus and Poisson’s ratio were 5% and 26%, respectively. Recently, Andreads et al. [25,26] proposed a novel embedded technique that uses E-glass fibres to cover the top of PZT transducers during lay-up to improve the mechanical properties of carbon fibre reinforced plastic (CFRP) composites. These mechanical results showed that their embedding technique had no effect on the integrity of the CFRP composites. Table 1 summarises the effects of embedded PZT transducers with different techniques on the mechanical properties of composites.

The authors’ previous study was focused on a novel circuit-printing technique using the edge cut-out method [13], which embedded printed diagnosed films with PZT transducers into composites. The use of the thin diagnosed films with printed circuits instead of traditional cables was shown to significantly reduce the weight of the composite structures. In addition, the novel edge cut-out method makes edge trimming possible, which can be used in industrial mass manufacturing for the next higher assembly. In this paper, the effect of fatigue tests on the integrity of the embedded PZT transducer is investigated. Furthermore, the reduction in the mechanical properties of the host composite structure is investigated through consideration of the worst-case scenario of the embedded film being embedded along the entire coupon. This is unlike the previous studies cited above, where the embedding only covered a small part of the host structure.

Initially, the effect of fatigue tests on the electro-mechanical impedance (EMI) properties of the PZT transducers under different loading cycles (up to 1 million) was studied. In addition, the effect of fatigue tests on the active sensing behaviours based on UGW under different loading cycles was investigated. Finally, tensile and compressive tests were conducted to assess the effects of the novel embedding technique on the elastic modulus of the composite materials.

## 2. Manufacturing

**Figure 1:**
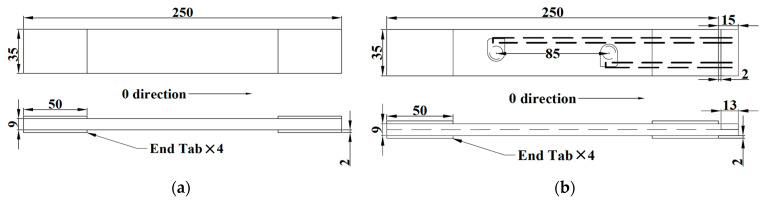
Schematic of drawings for (**a**) normal coupons and (**b**) coupons with embedded PZT transducers (unit: mm).

Hexply^®^ uni-directional carbon fibre prepregs IM7/8552 (Hexcel, Duxford, UK) were used and the quasi-isotropic stacking sequence for the lay-up was ((0°/+45°/−45°/+90°)_9_)_s_. The manufactured size of these coupons for machinal tests was according to the ASTM D695 standard. Figure 1a,b shows schematic drawings for the coupons without and with embedded PZT transducers, respectively. For the embedding, Kapton^®^ films (DuPont, Stevenage, UK) (DuPont^TM^ HN: 25 μm for the thickness) were used as the diagnosed films and the Dimatix printer (Fujifilm, Valhalla, NY, USA)(DMP-2580) was used to print circuits on these diagnosed films. DuraAct^TM^ PZT transducers (PI Ceramic GmbH, Lederhose, Germany)(P-876.K025) were connected to the printed circuits using mixed conductive epoxy resin/hardener agents (RS 186-3616). High temperature ending terminals (Techni Measure Ltd, Doncaster, UK)(TML Co. TPF-2M) were then connected to the other side of the printed circuits by applying the above conductive agents for convenient soldering and connecting the exposed wires without damaging the printed circuits after curing.

**Figure 2:**
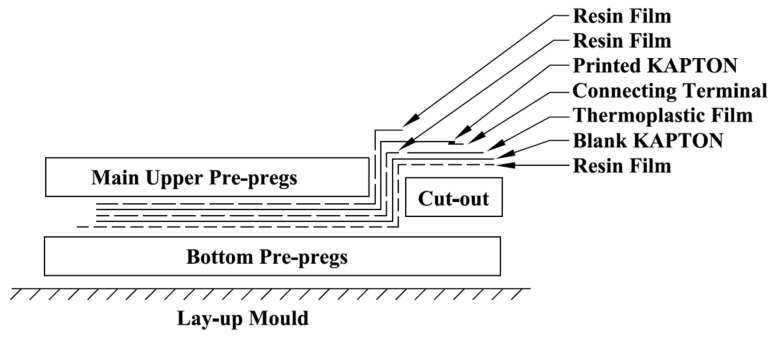
Schematic of embedding procedure during lay-up.

**Figure 3:**
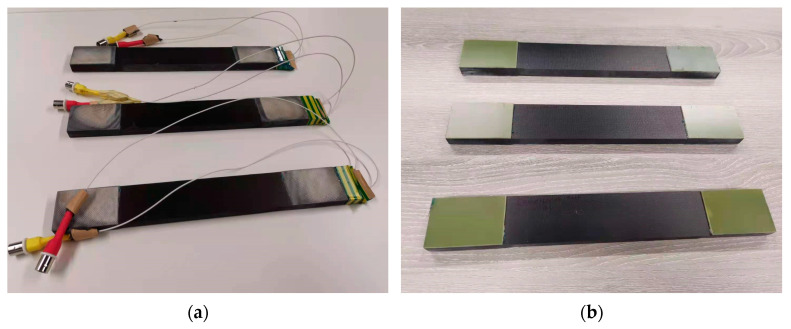
Examples of (**a**) coupons with embedded PZT transducers and (**b**) standard coupons.

Figure 2 illustrates the schematic of embedding procedures during lay-up based on a novel cut-out method. As is shown in Figure 2, resin films (Hexcel, Duxford, UK) (Hexply^®^ M21) were used to bond the prepregs and diagnosed films to increase the bonding quality and to prevent delimitations during the fabrication. In addition, two layers of thermoplastic films (Pontocal AG, Schmitten, Germany)(TPU-Pontocal AG) were placed under the ending terminals to prevent these terminals’ surfaces from being covered by melt resin films during the curing procedure due to their stable properties. The preparation of the printed diagnosed films and the details of the novel cut-out method for embedding during layup can be referred to in the authors’ previous work [13]. After lay-up, these carbon fibre prepregs were then cured at 180 °C in an autoclave: the curing cycle followed the specification of curing conditions from its datasheet. After finishing the fabrication, the wires were soldered to the ending terminals to connect the embedded diagnosed films with their printed circuits and PZT transducers. Figure 3a,b shows the pictures of the manufactured coupons with and without embedded PZT transducers, respectively.

## 3. Fatigue Tests

**Figure 4:**
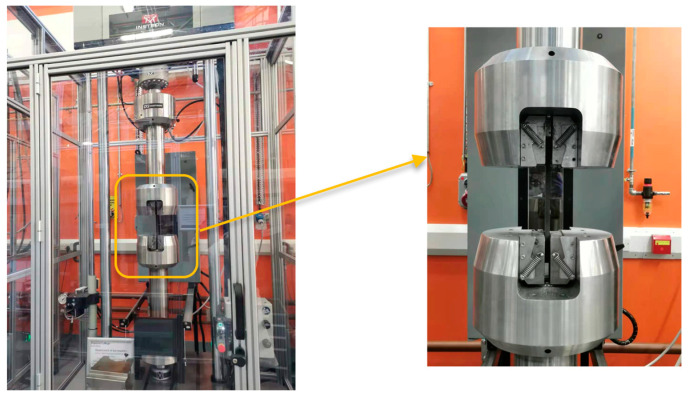
Schematic of Instron Hydraulic 250 kN test machine.

To study the effects of fatigue loading on the integrity of the bonding properties and sensing performance of the embedded sensors, tension–tension fatigue tests for the composite coupons with embedded PZT transducers were conducted. The EMI properties for the embedded PZT transducers and signal stabilities under different loading cycles (up to 1 million cycles) are presented in this section. An Instron Hydraulic 250 kN test machine was used for the fatigue tests, as shown in Figure 4. There were two scenarios for the force range in the fatigue tests: (a) 0.5~5 kN and (b) 1~10 kN (stress ratio of 0.1 for aeronautic research). Table 2 shows the test plan: the machine was stopped after each cycle step to allow for EMI and UGW data to be measured for each load range.

### 3.1. Electro-Mechanical Impedance (EMI) Properties

Electro-mechanical impedance (EMI) based on the converse piezoelectric effect that a strain is generated by applying a harmonic voltage for a PZT actuator [27]. The theory of the EMI method based on admittance measurements can be referred to in [13]. This method is one of the most promising methods in SHM technology, which can not only evaluate local damage severities but can also access transducers’ fractures, the degradation of mechanical/electrical properties of transducers, and the integrity of the bonding properties between the PZT transducer and its host structure [28,29]. It has been found that any change in host structure will change its vibration behaviours and the admittance of PZT transducers will be affected indirectly [30,31]. In addition, the change of the imaginary part of admittance at low frequency range will affect the integrity of the structure between the PZT transducer and its host structure [27].

**Figure 5:**
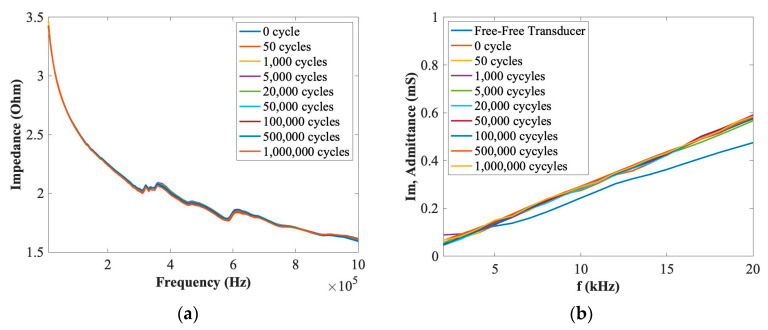
EMI properties of (**a**) impedance and (**b**) the imaginary part of admittance for the embedded PZT transducer under the force range of 0.5~5 kN.

**Figure 6:**
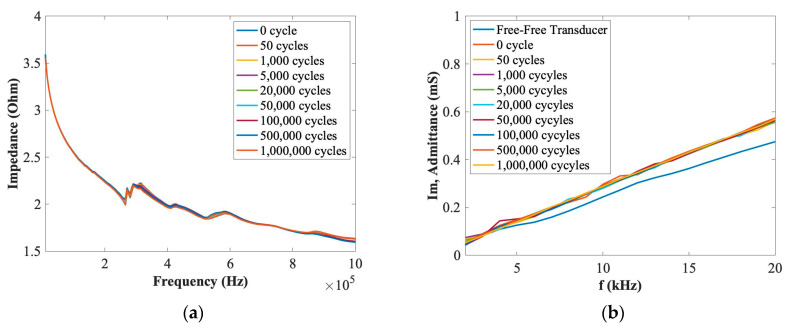
EMI properties of (**a**) impedance and (**b**) the imaginary part of admittance for the embedded PZT transducer under the force range of 1~10 kN.

In this section, the EMI method was used to investigate the integrity of bonding properties by comparing the slope difference of the imaginary part of admittance of PZT transducers at a low frequency range under different loading cycles. The aim was to investigate the effect of fatigue tests on the integrity of the bonding properties of the embedded PZT transducers after up to 1 million loading cycles. A KEYSIGHT Impedance Analyzer E4990A was used to measure the impedance value. A SinePhase Impedance Analyser (Model 16777 K) was used to measure the imaginary part of the admittance of a PZT transducer to compare differences between the embedded transducer under different loading cycles and its free–free situation. Figure 5 and Figure 6 show the EMI results of (a) the impedance and (b) the imaginary part of admittance at low frequency ranges for each PZT transducers for force ranges of 0.5~5 kN and 1~10 kN after 1 million cycles. As can be seen in these figures, there are no obvious differences for the impedance and the slope of the imaginary part of admittance at low frequency ranges after 1 million loading cyclesl therefore, the effect of the fatigue tests on the embedded PZT transducers can be ignored, and the stability and durability of these PZT transducers can be considered stable up to 1 million cycles.

### 3.2. Sensing Performance of PZT Transducers

To investigate the effect of fatigue tests on the sensing performance after 1 million loading cycles and to measure UGW signals, a National Instrument (NI) PXIe-1073 with a NI PXI-5412 arbitrary signal generator was used for signal generation, and a NI PXI-5105 digitizer was used to record UGW signals. Five-cycle Hanning-windowed tone burst signals at central frequencies of 50 kHz and 250 kHz according to the below equation were used as actuation signals, respectively [32], and the actuation amplitude and sampling frequency were 6 V and 100 MHz, respectively:

(1) $$f\left(t\right)=\frac{1}{2}V\sin\left(2\pi f_{c}t\right)\left[1-\cos\left(\frac{2\pi f_{c}t}{5}\right)\right]H\left(\frac{5}{f_{c}}-t\right)$$

where *V* is the actuation peak amplitude, *f_c_* is the central frequency, and *H* is the Heaviside step function. The stability of the UGW following tests with different loading cycles according to Table 2 was assessed.

#### 3.2.1. Fatigue Force Range 1: 0.5~5 kN

**Figure 7:**
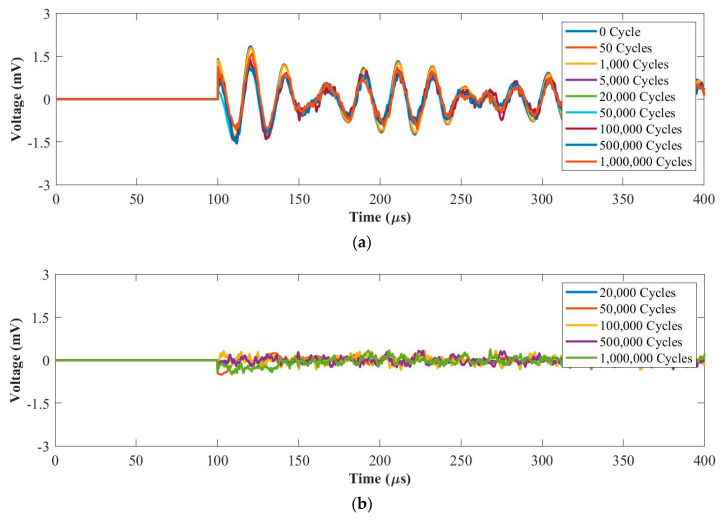
UGW comparisons within 1 million cycles for (**a**) original signals and (**b**) residual signals under the force range of 0.5~5 kN at 50 kHz.

**Figure 8:**
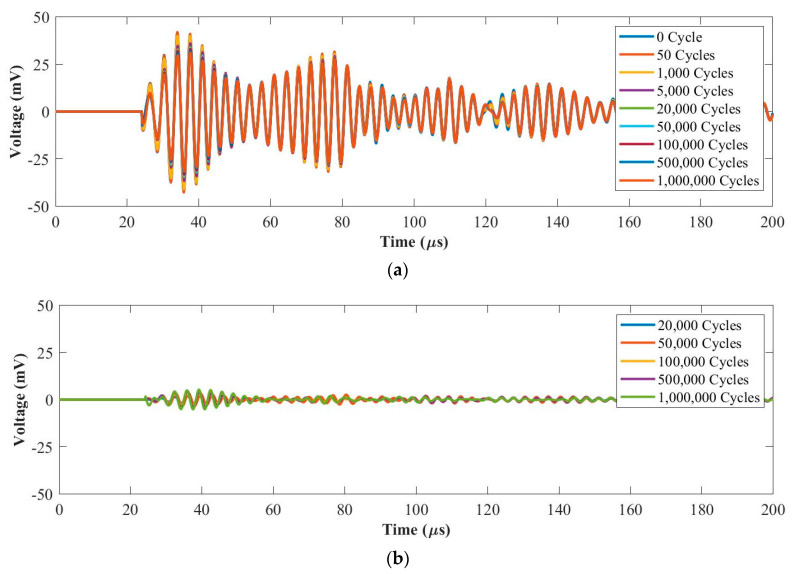
UGW comparisons within 1 million cycles for (**a**) original signals and (**b**) residual signals under the force range of 0.5~5 kN at 250 kHz.

Figure 7a and Figure 8a plot UGW signals after each fatigue cycle at 50 kHz and 250 kHz, respectively. Figure 7b and Figure 8b plot the residual signals (the difference between the current and baseline signal) from 20,000 cycles to 1 million cycles at 50 kHz and 250 kHz, respectively. In Figure 7a, the signals up to 1 million cycles maintain the same phase with amplitude reduction. This is due to the internal delamination generated in the composite coupon during the fatigue test. As can be seen in Figure 7b, the amplitudes of the residual signals maintain at the noise level from 20,000 cycles up to 1 million cycles. Therefore, the sensing performance remained good after 1 million loading cycles at 50 kHz.

As can be seen in Figure 8a, the amplitude of the first wave packet reduces after 1 million cycles due to the internal delamination generated in the composite coupon during the fatigue test. Figure 8b shows that the first wave packets of the residual signals were almost the same from 20,000 cycles to 1 million cycles at 250 kHz. Therefore, the sensing performance for fatigue tests under the force range of 0.5~5 kN is stable at 50 kHz and 250 kHz.

#### 3.2.2. Fatigue Force Range 2: 1~10 kN

**Figure 9:**
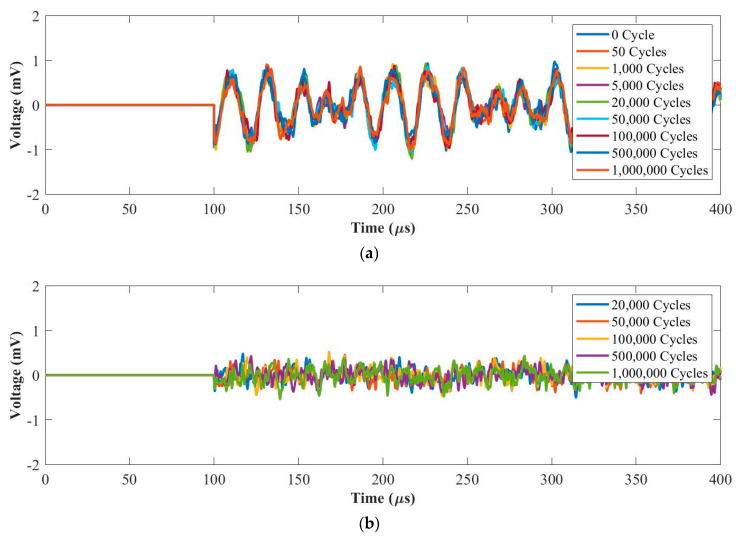
UGW comparisons within 1 million cycles for (**a**) original signals and (**b**) residual signals under the force range of 1~10 kN at 50 kHz.

**Figure 10:**
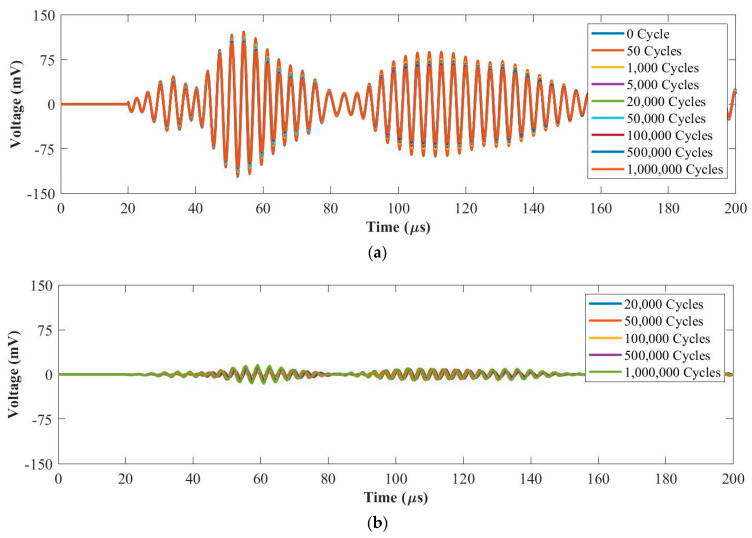
UGW comparisons within 1 million cycles for (**a**) original signals and (**b**) residual signals under the force range of 1~10 kN at 250 kHz.

Figure 9a and Figure 10a plot original UGW signals after each fatigue cycle at 50 kHz and 250 kHz, respectively. Figure 9b and Figure 10b plot the residual signals (the difference between the current and baseline signal) from 20,000 cycles to 1 million cycles at 50 kHz and 250 kHz, respectively. As can be seen in Figure 9, there are no obvious differences from the original UGW signals and the residual signals maintain at noise level from 20,000 cycles to 1 million cycles at 50 kHz. In Figure 10, the amplitude of the first wave packet of the original UGW signals reduces slightly due to the internal delamination generated in the composite coupon during the fatigue test. In addition, the residual signals are almost the same from 20,000 cycles to 1 million cycles at 250 kHz. Hence, the UGW for the fatigue tests under the load range of 1~10 kN are stable at both 50 kHz and 250 kHz frequencies.

## 4. Tensile and Compressive Tests

**Figure 11:**
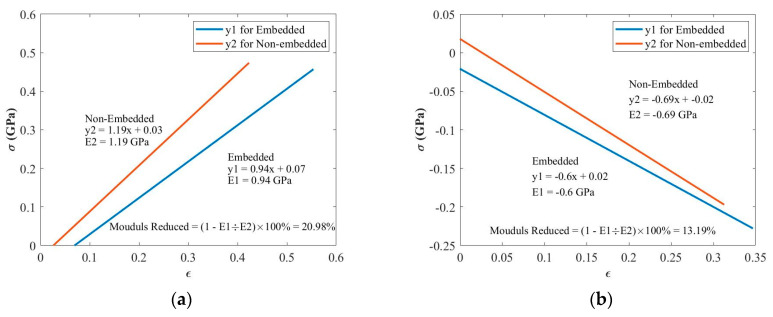
Mechanical properties studies for (**a**) tensile and (**b**) compressive modulus reductions compared to composite coupons without embedded PZT transducers.

To investigate the effect of the embedding technique on the elastic modulus of composites, tensile and compressive tests were conducted by comparing the difference between the coupons with and without embedded PZT transducers. An Instron Hydraulic 250 kN test machine was used for the tensile and compressive tests, as shown in Figure 4. For setting up the parameters of the tensile and compressive tests, the duration of the ramp for the waveform was 1.5 min, and the ending point was 200 kN for tensile tests and −80 kN for compressive tests. Figure 11a,b plots the stress–strain curve before the failure between the coupons with and without embedded PZT transducers for the tensile and compressive tests, respectively. The elastic modulus is the slope of the curve computed by the polyfit function in MATLAB during the post-processing procedure.

As can be seen in Figure 11, the elastic modulus was reduced by about 20.98% for the tensile tests and 13.19% for the compressive tests. Although the elastic modulus for the tensile/compressive tests reduced more in the present work than in other authors’ work, the case presented for these coupons is the worst possible case, which is impractical for manufacturing real composite components with embedded diagnosed films with PZT transducers for SHM purposes. Considering other authors’ work, they only embedded the small area of different types of sensors, while in this case an entire layer was completely embedded across the entire coupon due to its limited size. In addition, due to the surface toughness of Kapton films and the shear reduction between the prepreg plies, the elastic modulus would reduce more if only a small strip of film was embedded in the centre of the coupon rather than the entire diagnosed film being embedded though the middle ply. Furthermore, the thickness of the coupon may also affect the results. The thinner thickness in other authors’ work compared to this case (9 mm) would also improve their work. Therefore, this level of reduction is considered reasonable given the fact that the diagnostic film was embedded over the entire surface of the text specimen.

## 5. Conclusions

In this paper, mechanical tests for composite coupons embedded with PZT transducers were investigated. First, fatigue tests for the force ranges of 0.5~5 kN and 1~10 kN with 1 million cycles were studied. The stability of the EMI properties and active sensing behaviours of UGW remained good up to 1 million cycles at 50 kHz and 250 kHz. It was demonstrated that the novel embedded circuits printed on the diagnosed film remained conductive and the embedded PZT transducers remained active and intact after 1 million loading cycles, which indicates the embedded diagnosed film with networks of PZT transducers can withstand the mechanical loads applied to it.

Second, for tensile and compressive tests, the reduction of the elastic modulus between the standard coupons and the coupons embedded with the diagnosed film with PZT transducers were compared. It was found that the embedded diagnosed film with PZT transducers caused the tensile modulus to reduce by about 20.98% and the compressive modulus to reduce by about 13.19%. Although the strength reduction is not ideal for this situation, the anticipated results would be significantly less when reducing the unprinted area of the diagnosed film during lay-up. However, considering the benefits of this novel embedding technique, the embedded diagnosed film with networks of PZT transducers can be applied as a layer (prepreg) during lay-up. In addition, this technique allows for the embedding of large sensor networks with an optimizing design of connections. Therefore, this embedding technique is a promising method for SHM applications.

## Figures and Tables

**Table 1:** Summary of effects of embedded PZT transducers on mechanical properties for composites.

No.	Embedding Technique	Effects of Embedding on Different Mechanical Tests
1	SMART Layers	Did not degrade for mechanical properties when embedded in a relatively small section of the part.
2	PZT with wires	Mechanical resistance and longitudinal strain 6% and 11% ↑; Elastic modulus and Poisson’s ratio 5% and 26% ↓.
3	PZT with wires	Using E-glass to cover embedded sensors: no effects on the integrity for mechanical properties.
4	Diagnosed film with PZT	Fatigue test: EMI and sensing performance remained good. Tensile and compressive modules: 20.98% and 13.19% ↓ when the diagnosed film embedded across the entire layer.

**Table 2:** Fatigue test plan.

No.	Loading Cycles
1	0
2	50
3	1000
4	5000
5	20,000
6	50,000
7	100,000
8	500,000
9	1,000,000

## Data Availability

Data Sharing is not applicable.
